# Modeling of PTPN22 and HLA-DRB1 susceptibility to rheumatoid arthritis

**DOI:** 10.1186/1753-6561-1-s1-s14

**Published:** 2007-12-18

**Authors:** France Gagnon, David Hajage, Sabine Plancoulaine, Sophie Tezenas du Montcel

**Affiliations:** 1 Department of Public Health Sciences, Faculty of Medicine, University of Toronto, 155 College Street, Toronto, Ontario, M5T 3M7, Canada; 2 Université Pierre et Marie Curie-Paris 6, EA3974 Modélisation en Recherche Clinique, Paris, F-75013, France, AP-HP, Groupe Hospitalier Pitié-Salpétrière, Département de Santé Publique, Unité de Biostatistique et Information Médicale, Paris, F-75013, France; 3 INSERM, U550, Paris, F-75015, France; Laboratoire de Génétique Humaine des Maladies Infectieuses, Faculté de Médecine René Descartes, Université de Paris René Descartes, site Necker, Paris, F-75015, France

## Abstract

In the present paper, we used the North American Rheumatoid Arthritis Consortium data provided for Genetic Analysis Workshop 15 Problem 2 to: 1) estimate the penetrances of PTPN22 and HLA-DRB1 and, 2) test the selected model of PTPN22 conditional on the rheumatoid factor status. To achieve these aims, we used the marker association segregation chi-square method, fitting simultaneously both genotype frequency and identical by descent distributions in a sample of 3690 White individuals from 604 nuclear families. A co-dominant model fitted the rs2476601 (R620W) single-nucleotide polymorphism (SNP) of the PTPN22 gene well, whereas a lack of fit for all models was observed for the HLA-DRB1 locus. Testing genetic models of rheumatoid arthritis that include the PTPN22 SNP in addition to the HLA-DRB1 locus did not affect the results, nor did subgroup analysis of PTPN22 conditional on the rheumatoid factor status. In conclusion, PTPN22 R620W SNP is a risk factor for rheumatoid arthritis. The genetic architecture of the HLA-DRB1 locus is highly complex, and more elaborate modeling of this locus is required.

## Background

The HLA locus, and in particular several alleles of HLA-DRB1, have been associated with rheumatoid arthritis (RA) and other autoimmune disorders [1,2]. The specific alleles being implicated vary according to population. Commonly reported set of alleles are DRB1*0101, 0102, 0401, 0404, 0405, 0408, and 1001 [2]. However, these alleles are either associated with severe forms of RA, or are weakly and sometime inconsistently associated with RA. Moreover, variability in disease expression is observed in individuals with the same HLA background, ranging from some being severely affected to others being unaffected [2]. Therefore, HLA-DRB1 does not alone explain the genetic susceptibility to common forms of RA. In addition to the HLA locus, other regions have been identified by genome scans [1,3,4] and/or by candidate gene approaches [1,5]. Among the non-HLA loci, PTPN22, a gene encoding for protein tyrosine phosphatase non-receptor 22, is considered a strong candidate [1]. Phosphatases are known to play a role in immune-cell homeostasis. Recently, a functional single-nucleotide polymorphism (SNP) in PTPN22 gene (R620W allele in rs2476601) was reported to be associated with RA [4,5]. However, neither HLA-DRB1 nor PTPN22 rs 2476601 individually fully explain the genetic contribution to RA, nor they are necessary or sufficient for RA to be present in a given individual. Moreover, the association to RA may be specific to rheumatoid factor-positive RA patients, as recently reported for another PTPN22 variant [6]. Because there is strong evidence implicating these two genes in RA, and because the effect of PTPN22 variants may be restricted to rheumatoid factor-positive patients, the purpose of our study is twofold: first, to estimate the genetic model (including penetrances) associated with these two genes in White nuclear families from the North American Rheumatoid Arthritis Consortium (NARAC) data; and second, to model PTPN22 susceptibility conditional on the rheumatoid factor status in RA patients. In the present paper, we report that a co-dominant model best fits the PTPN22 data and that stratification based on rheumatoid factor status did not modified the results. No model fitted the HLA-DRB1 locus.

## Methods

### Data

We used the NARAC data of Problem 2 of the Genetic Analysis Workshop 15 (GAW15). For the present paper, only the data from individuals labeled as "Caucasian", and those with "unknown" ethnicity, were considered (*n *= 3690). We pooled these two ethnic categories because the majority of the NARAC sample was white and the allele frequencies for the four HLA-DRB1 microsatellites (d6S265, TNFA, D6S273, and D6S1629), as well as the rs2476601 SNP for PTPN22, were not statistically different in the "unknown" ethnicity group compared to the one labeled as Caucasian. We selected a sib-pair design approach, and only one sib-pair per nuclear family was included in the analysis. We could not use the full sib-pair data available with the selected analytic approach due to the non-independence of the various sib-pairs within sibships. In families with more than two affected sibs, the sibs with the most complete data and the closest in age were those kept for analysis. The remaining affected sibs, half sibs, and unaffected sibs, were excluded from the analysis. In the context of multigenerational pedigrees, each generation was split into nuclear families. Parental data were used to compute the penetrances. Using the data provided through GAW15, we defined as an index case the sib from each pair that had the most complete data.

### HLA-DRB1 allele classification and PTPN22 rs2476601 allele labeling

Because the HLA-DRB1 alleles reported to be associated with RA share an RAA motif at position 71–74, our classification of the HLA-DRB1 alleles is based on their amino acid sequence at that position [7]. When allele sub-types were not available, we assigned the allele according to their frequencies (e.g., 01R alleles were considered as *0101; individuals with HLA-DRB*14 alleles were randomly assigned half to *1401 and half *1402). We assigned the HLA-DRB1*0101, *0405, *0408, *1001, *1402, and *16 as E1, *0401 and *0409 as E2, *0102, *0404, *423, *12, and *1406 as E3, and the other alleles as Ex. The alleles classified as Ex were considered as the non-susceptibility alleles. For PTPN22, the susceptibility allele of the R620W missense SNP (rs2476601) corresponds to the minor allele T, whereas the common allele is C.

### Statistical analyses

Genetic models for HLA-DRB1 and PTPN22 genes in RA were tested using the marker association segregation chi-square (MASC) method [8]. Details of this approach have been described elsewhere [7,8]. Briefly, the MASC method is based on the idea of minimizing a sum of independent chi-squares and testing the goodness-of-fit of various models [8]. This approach estimates penetrances simultaneously using information on the marker association and segregation with the disease. Thus, MASC uses the allelic association information from the genotype distribution among unrelated index cases, as well as the linkage information, based on the proportion of siblings sharing 2, 1, or 0 alleles identical by descent (IBD), from each index case and its affected sib. To deal with potential IBD estimation uncertainty, the probability of sharing 2, 1, or 0 alleles IBD was computed for flanking SNPs using MERLIN. Based on IBD estimation probability of equal or more than 80% for individual SNP, no ambiguity in IBD sharing information was detected. Because the MASC approach at some point is conditioning on the IBD status, it cannot accommodate analyses of large sibships due the non-independence of the sib-pairs within sibships. Nuclear families with the following three configurations were included: affected sibs and unaffected parents, affected sibs and one affected parent, and affected sibs and affected parents. These corresponded to MASC labels of C_2_, C_4_, and C_6_, respectively. The data was then stratified according to these distributions (i.e., family, genotype, and IBD distributions) using the information on both the index cases and their relatives. The expected distributions were then computed for each proposed model. These distributions were used to estimate the relative penetrance of each genotype, which are the ratio of the penetrance for a given genotype to the penetrance for the referent (i.e., the higher-risk genotype). These estimations require knowledge of the genotype frequencies in the general population. These were estimated using the affected family-based association method (AFBAC) [9]. This approach uses the parental alleles not transmitted to the children, assuming Hardy-Weinberg equilibrium. In the context of multiplex ascertainment, such as in the current study, the average of the parental alleles transmitted to both sibs is compared to the AFBAC population of parental alleles never transmitted to the affected sib-pair. In the framework of MASC, the genetic model is good (i.e., explains the observed association and linkage data) when the expected and the observed distributions do not differ significantly. Therefore, a *p*-value > 0.05 will correspond to the acceptance of the model, whereas a *p*-value < 0.05 implies that other factors not modeled are involved in the disease expression. We first fitted the co-dominant model, i.e., the most general model. To test whether the penetrance of the different genotypes differ significantly, pairwise comparisons were performed with the maximum likelihood ratio test. Since the co-dominant model fitted well, we then tested if the penetrance for the heterozygotes and the homozygotes were equals (dominant vs. co-dominant, recessive vs. co-dominant). Confidence intervals for all estimates were computed using a bootstrap procedure.

## Results and discussion

### Lack of fit of IBD data for HLA-DRB1 (data not shown)

The estimated general population allele frequencies are 15%, 6%, 5%, and 74% for E_1_, E_2_, E_3_, and E_x_, respectively. These frequencies are similar to those of the population recruited in France through the European Consortium on Rheumatoid Arthritis [7]. However, the allele classification used in our study was not exactly like the new classification validated by the European Consortium study because of the lack of sub-typing for some alleles, in particular DRB1*11 and DRB1*13. In our study, all the expected distributions related to HLA-DRB1 differed significantly from the observed distributions (*p *= 0.002). In particular, in C_4 _families (i.e., the sibs and one parent are affected), 79% of E_x_/E_x _probands share one HLA-DRB1 allele IBD with their affected sibling, compared to the expected proportion of 48%. Thus, this model does not explain all the observations. Therefore, we could not model epistasis analyzing HLA-DRB1 conditional on PTPN22.

### A co-dominant model fits IBD data for *PTPN22*

The estimated general population allele frequency for the T allele is 5% (thus, 95% for C) (data not shown). Table 1 shows the expected and the observed distributions for the family configurations, the genotypes, and the IBD sharing. Compared to the distributions at the HLA-DRB1 locus, there are more missing genotype values for the PTPN22 locus, in particular for parents. Moreover, determination of IBD is sometimes difficult due to the bi-allelic nature of the rs2476601 marker (i.e., SNP). Table 2 presents the penetrances and coupling estimated under various models. Only the co-dominant model fits the observed distributions. Estimated penetrances for the co-dominant model are 0.54 and 0.20 for TC and CC respectively, versus the fixed penetrance of 1 for TT. The dominant model with an estimated penetrance of 0.20 for CC compared to TC and TT combined was rejected, as well as the recessive model with a penetrance of 0.17 for TC and CC combined compared to TT. Confidence intervals on the penetrances, based on a bootstrap approach, are presented. The *p*-values are 0.20, 0.02, and <0.0001 for the co-dominant, dominant, and recessive models, respectively. The global penetrance, i.e., the estimated penetrance for TT, is 0.37. In Table 2, coupling refers to the probability of carrying the susceptibility allele knowing the marker allele. In this table, we observe that the T allele has a probability of 1, whereas the probability associated with the C allele is much lower (0.07). This strongly suggests that the susceptibility allele is T, i.e., the marker allele, or that of a gene in very close linkage disequilibrium with the marker locus.

**Table 1:** Expected^a ^(Exp.) and Observed (Obs.) family configurations, genotypes and stratified IBD distributions for PTPN22 rs2476601

				IBD distribution (%)					
				
		Genotype distribution (%)		IBD = 2		IBD = 1		IBD = 0	
					
Family configuration^b^	Genotype	Exp.	Obs.	Exp.	Obs.	Exp.	Obs.	Exp.	Obs.
C_2_	TT	1	2	42	25	47	75	11	0
n_f _= 447 Obs.	TC	24	24	34	25	50	48	16	27
n_f _= 449 Exp.	CC	75	74	26	22	50	55	24	23
									
C_4_	TT	3	2	-	50	-	50	-	0
n_f _= 147 Obs.	TC	31	32	33	22	50	61	17	17
n_f _= 144 Exp.	CC	66	66	26	36	50	41	24	23
									
C_6_	TT	5	0	-	-	-	-	-	-
n_f _= 10 Obs.	TC	35	30	-	50	-	50	-	0
n_f _= 11 Exp.	CC	60	70	-	0	-	50	-	50

^a^Expected distributions are given under the co-dominant model.

^b^Family configurations: C_2_, sibs are affected; parents are unaffected; C_4_, sibs are affected; one parent is affected; C_6_, sibs and parents are affected;

n_f_, number of families.

-, not computed. Genotype distributions for which the sample size is ten or less, and IBD distributions for which the sample size is five or less are not include in the model.

**Table 2:** Penetrance and coupling associated with PTPN22 rs2476601

Genotype	Co-dominant	Dominant model	Recessive model
Penetrance^a^			
TT	1	1	1
TC	0.54 [0.42–0.75]	1	0.17 [0.13–0.22]
CC	0.20 [0.12–0.30]	0.20 [0.14–0.28]	0.17 [0.13–0.22]
			
Coupling			
T	0.99 [0.54–1]	0.27 [0.15–0.43]	0.99 [0.96–1]
C	0.07 [0–0.13]	0.01 [0–0.02]	0.22 [0.19–0.28]
			
-2*log likelihood	-2066.63	-2073.72	-2093.58
*p*-value	0.20	0.02	1.5 × 10^-5^

^a^The reference genotype is TT

### Testing the co-dominant model of PTPN22 R620W conditional on the rheumatoid factor status

Table 3 shows the penetrance and coupling associated to PTPN22 stratified according to the rheumatoid factor (RF) status of the index case. RF+ and RF- are defined by IgM RF values >14 and ≤14, respectively. For this analysis, 3648 individuals had known RF status (3002 RF+ and 646 RF-) from 596 families (489 RF+ and 107 RF-). For both RF+ and RF-, models fit with the observed distribution (*p *= 0.33 for RF+, and 0.23 for RF-). However, the difference between the stratified and unstratified models was not statistically significant (χ^2 ^= 2.816, df = 4, *p *= 0.589).

**Table 3:** Penetrance and coupling associated with PTPN22 stratified by rheumatoid factor (RF) status

	Patient status		
		
Genotype	RF+	RF-	Total sample
Penetrance^a^			
TT	1	1	1
TC	0.55	0.34	0.52
CC	0.19	0.20	0.19
			
Coupling			
T	0.96	0.99	0.97
C	0.07	0.13	0.07
			
-2*log likelihood	1677.152	367.36	2047.328
*p*-value	0.33	0.23	0.20

^a^The global penetrance is set to 0.37 in all models. The reference genotype is TT.

## Conclusion

Our results support the role of SNP R620W of the PTPN22 gene in the risk of developing RA. None of the models tested fitted the data for HLA-DRB1. This further reinforce the conclusion of Tezenas du Montcel et al. [7] that classification of HLA-DRB1 alleles is complex, and that some classification systems may not capture the complexity at this locus. Alternatively, the lack of fit of the main models tested for HLA-DRB1 may also suggest that epistasis is a major part of the underlying genetic architecture for this locus, i.e., effect of an interaction without measurable main effects. Better understanding of the genetic architecture of RA will be essential not only to identify additional genes implicated in RA but also as a critical component to an eventual translation of genetic research knowledge into clinical benefits for patients.

## Competing interests

The author(s) declare that they have no competing interests.
